# Getting Ready for the Hydrogen Evolution Reaction: The Infrared Spectrum of Hydrated Aluminum Hydride–Hydroxide HAlOH^+^(H_2_O)_*n*−1_, *n=*9–14

**DOI:** 10.1002/anie.202105166

**Published:** 2021-06-04

**Authors:** Jakob Heller, Wai Kit Tang, Ethan M. Cunningham, Ephrem G. Demissie, Christian van der Linde, Wing Ka Lam, Milan Ončák, Chi‐Kit Siu, Martin K. Beyer

**Affiliations:** ^1^ Institut für Ionenphysik und Angewandte Physik Universität Innsbruck Technikerstrasse 25 6020 Innsbruck Austria; ^2^ Department of Chemistry City University of Hong Kong 83 Tat Chee Avenue Kowloon Tong Hong Kong SAR P.R. China

**Keywords:** hydrogen bonding network, metal hydride, proton transfer, vibrational spectroscopy, water splitting

## Abstract

Hydrated singly charged aluminum ions eliminate molecular hydrogen in a size regime from 11 to 24 water molecules. Here we probe the structure of HAlOH^+^(H_2_O)_n−1_, n=9–14, by infrared multiple photon spectroscopy in the region of 1400–2250 cm^−1^. Based on quantum chemical calculations, we assign the features at 1940 cm^−1^ and 1850 cm^−1^ to the Al−H stretch in five‐ and six‐coordinate aluminum(III) complexes, respectively. Hydrogen bonding towards the hydride is observed, starting at n=12. The frequency of the Al−H stretch is very sensitive to the structure of the hydrogen bonding network, and the large number of isomers leads to significant broadening and red‐shifting of the absorption of the hydrogen‐bonded Al−H stretch. The hydride can even act as a double hydrogen bond acceptor, shifting the Al−H stretch to frequencies below those of the water bending mode. The onset of hydrogen bonding and disappearance of the free Al−H stretch coincides with the onset of hydrogen evolution.

## Graphical abstract

Water molecules must arrange around an aluminum hydride–hydroxide unit in a specific way to enable elimination of molecular hydrogen. Infrared multiple photon dissociation spectroscopy reveals that hydrogen bonding to the hydride takes place in a six‐coordinate Al^III^ hydride–hydroxide complex. The smallest cluster size for which hydride hydrogen bonding dominates is also the cluster size for which hydrogen evolution becomes efficient.

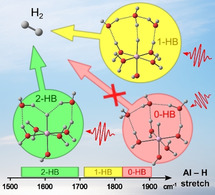

The hydrogen evolution reaction (HER) is key to storage of excess renewable energy via water electrolysis,^[1]^ as well as direct light harvesting by photocatalysts.^[2]^ In electrochemical HER studies, usually the net half reaction at the cathode is reported, with two protons recombining with two electrons forming H_2_. Regarding the reaction mechanism, two pathways are conceivable: hydrogen evolution may take place via recombination of two surface‐adsorbed hydrogen atoms^[3]^ or via hydride–proton recombination.^[4]^ Formation of the H_2_ molecule from two free H atoms is energetically demanding and does not play a role in practical processes. The mechanistic details are of utmost relevance to the development of novel electrocatalysts and efficient electrolyzers.

Hydrated metal ions in the gas phase are important model systems to study hydrogen evolution reactions at a molecular level. In gas‐phase clusters, several systems show hydrogen evolution upon exposure to room‐temperature black‐body radiation,^[5]^ in particular Mg^+^(H_2_O)_*n*_,^[6]^ Al^+^(H_2_O)_*n*_,[^7^, ^8^] and V^+^(H_2_O)_*n*_.^[9]^ Photochemical hydrogen formation has also been studied for Mg^+^(H_2_O)_*n*_ ^[10]^ and V^+^(H_2_O)_*n*_.^[11]^ The formation of H_2_ from Al^+^(H_2_O)_*n*_ activated by black‐body radiation exhibits an intriguing size dependence,[^7^, ^8^] which gave some hints on possible mechanisms. Quantum chemical calculations by Reinhard and Niedner‐Schatteburg^[12]^ along with ab initio molecular dynamics simulations by Siu and Liu^[13]^ revealed that the reaction takes place in two steps: First, a concerted proton transfer takes place through a water “wire” of at least three H_2_O molecules, from a first‐shell water molecule to the other side of the Al^+^ center, where the proton is reduced to hydride and simultaneously Al^I^ is oxidized to Al^III^. This leads to formation of a hydrated hydride–hydroxide complex, HAlOH^+^(H_2_O)_*n*−1_. This insertion reaction was already modeled quantum chemically by Watanabe and Iwata in 1995.^[14]^ A second proton transfer from a first‐shell water molecule, again through a water wire connected to the hydride serving as a hydrogen bond acceptor, leads to H_2_ formation together with Al(OH)_2_^+^(H_2_O)_*n*−2_. So far, the only indirect experimental evidence for this mechanism is an H_2_O/D_2_O exchange experiment, revealing that proton transfer takes place in Al^+^(H_2_O)_*n*_,^[15]^ which supports the presence of the HAlOH^+^(H_2_O)_*n*−1_ hydride–hydroxide structure. However, it still remains unclear whether the hydrogen bond towards the hydride really exists, and whether this structural feature is stable for a substantial amount of time or immediately leads to H_2_ elimination.

Here we studied the spectroscopy of gas‐phase hydrated aluminum ions Al^+^(H_2_O)_*n*_, *n=*9–14, by infrared multiple photon dissociation spectroscopy (IRMPD)^[16]^ in the 1400–2250 cm^−1^ region. The ions were generated in a laser vaporization source^[17]^ and stored in an ICR cell which is cooled to approximately 85 K, minimizing the influence of black‐body radiation.^[18]^ Cluster ions were irradiated with light from a tunable optical‐parametric oscillator (OPO) system operated at a pulse frequency of 1000 Hz, which amounted to quasi‐continuous irradiation on the timescale of the ICR experiment. The cluster size of interest was mass‐selected by resonant excitation of unwanted ions, irradiated for 0.2 s, and a mass spectrum was recorded. This procedure was repeated 15 times for each infrared wavenumber to improve the signal‐to‐noise ratio. Photon absorption led to evaporation of water molecules, and in some cases H_2_ elimination. The fragment intensity was quantified by mass spectrometry. Typical mass spectra are shown in Figure S2.

Figure 1 shows the IRMPD spectra for *n=*9–14. In this size regime, theory predicts the HAlOH^+^(H_2_O)_*n*−1_ structure,[^12^, ^13^] and *n=*11 is the smallest cluster for which H_2_ evolution was reported.[^7^, ^8^] In the *n=*9 spectrum, the prominent band at 1610 cm^−1^ is assigned to the water bending mode, while the band at 1940 cm^−1^ lies close to the *ν*_3_ mode (Al−H stretch) of AlH_3_ reported by Andrews and co‐workers,^[19]^ confirming the hydride–hydroxide structure. At *n=*10, a red‐shifted band around 1870 cm^−1^ emerges, which indicates the co‐existence of two chemically distinct aluminum hydride species. At *n=*11, the feature at 1940 cm^−1^ has almost disappeared, and the water bending mode region broadens. For *n=*12, H_2_ evolution sets in, triggered by infrared radiation, and the remaining feature in the aluminum hydride stretching region shifts to 1850 cm^−1^. Loss of H_2_+*x* H_2_O, *x=*2, 3, is as intense as H_2_O loss at *n=*13, and the Al−H stretch region broadens considerably and shifts to the red. At the same time, the blue shoulder in the H_2_O bending region loses intensity. This trend is more pronounced for *n=*14, while H_2_ evolution plays a smaller role than for *n=*13. H_2_ formation is accompanied by evaporation of two to three water molecules due to the exothermicity of the reaction, consistent with the earlier black‐body infrared radiative dissociation (BIRD) experiments[^7^, ^8^] as well as theory.[^12^, ^13^]

**Figure 1:**
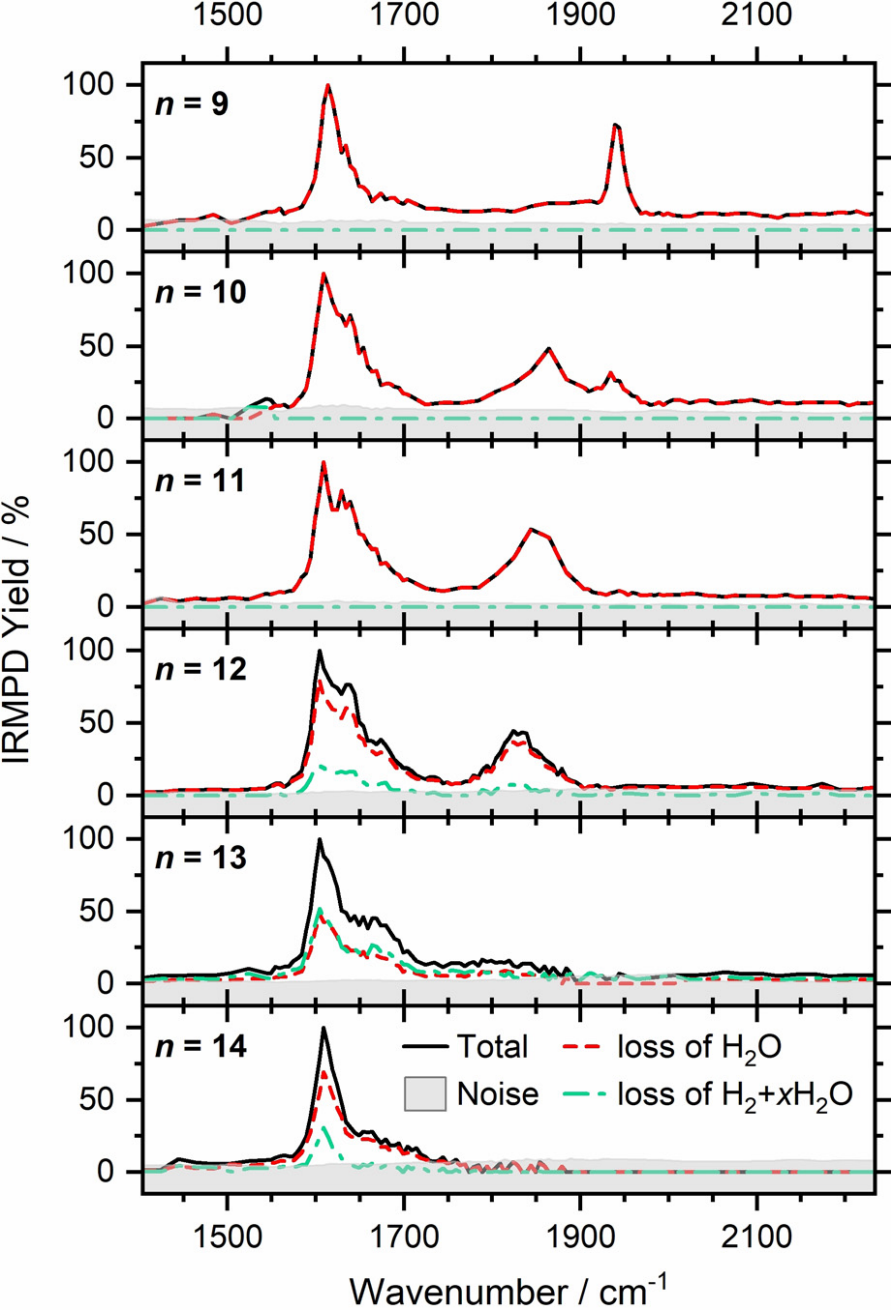
Experimental IRMPD spectra of Al^+^(H_2_O)_*n*_, *n=*9–14.

Quantum chemical calculations at the B3LYP/6‐311++G** level of theory were performed using the Gaussian software package.^[20]^ The infrared spectra of energetically low‐lying structures were simulated by applying a scaling factor of 0.982 and a Gaussian broadening with 20 cm^−1^ full‐width‐at‐half‐maximum to the harmonic frequencies.^[21]^ Energies were evaluated at the M06/6‐311++G** level of theory after re‐optimization of the respective geometries; while the M06 functional may predict unrealistic variations of absorption intensities,^[22]^ it is known to provide more reliable energies for hydrogen‐bonded systems than B3LYP.^[23]^ All reported energies are zero‐point corrected, using harmonic frequencies without scaling. Figure 2 shows the lowest‐energy structures found in our extensive search, additional structures are provided in the SI. Four‐, five‐, and six‐coordinate HAlOH^+^(H_2_O)_*n*−1_ complexes were investigated, denoted ***n*** **‐4 c**, ***n*** **‐5 c**, and ***n*** **‐6 c**, respectively. For *n*≥11, hydrogen bonding to the hydride becomes energetically competitive, and complexes with one and two hydrogen bonds towards the hydride are labeled ***n*** **‐6 c‐HB** and ***n*** **‐6 c‐HB2**, respectively.

**Figure 2:**
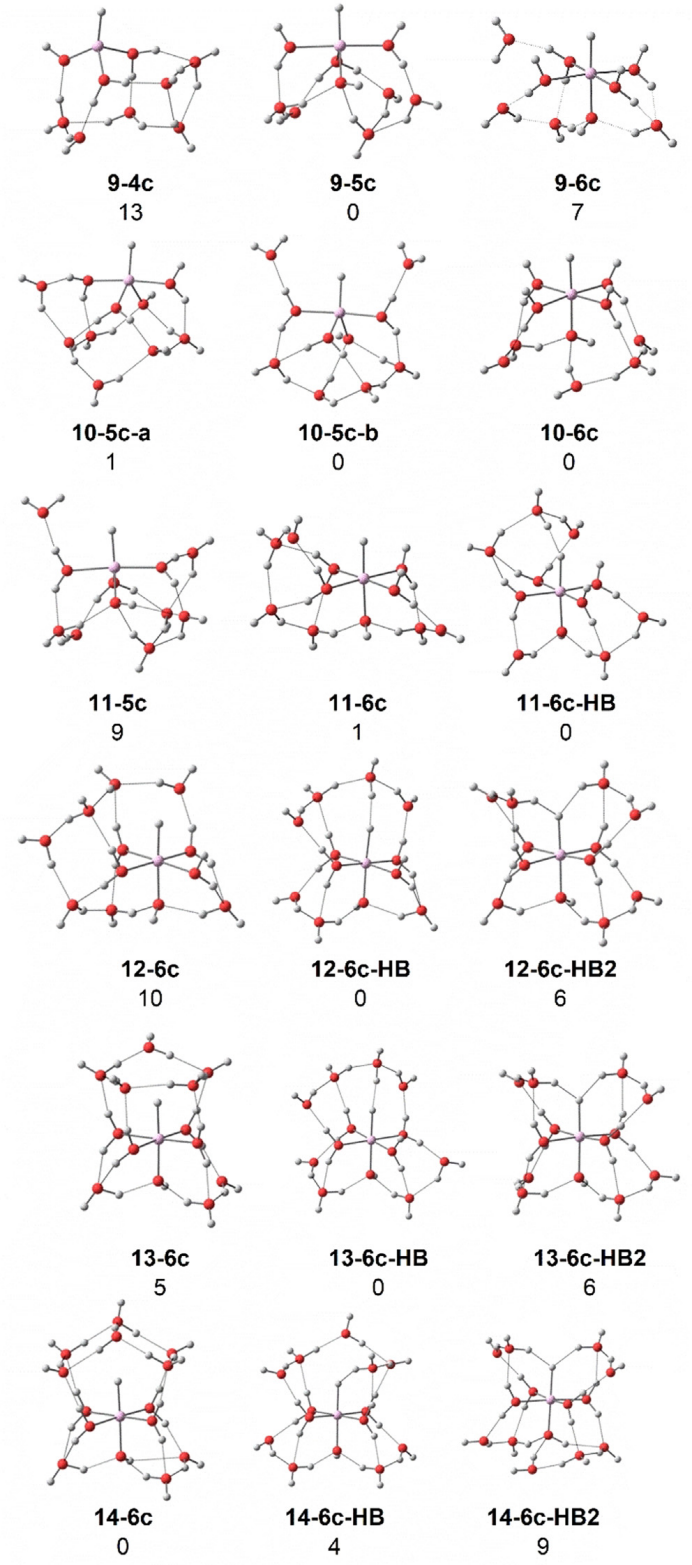
Selected low‐energy structures of HAlOH^+^(H_2_O)_*n*‐1_ for *n=*9–14. Relative energies at 0 K in kJ mol^−1^ were evaluated at the M06/6‐311++G** level of theory.

In Figure 3 we compare the simulated spectra of these low‐lying structures with the experimental IRMPD spectra. For *n=*9, the five‐coordinate complex **9‐5 c** exhibits a free Al−H stretch that closely matches the experimental band position. It is also the energetically lowest‐lying structure we found, and the position of the main water bending absorption is well reproduced. However, the calculated spectra exhibit a pronounced structure in the water‐bending region, while the experiment shows substantial broadening. We attribute this to the presence of a wide variety of isomers, which is typical for hydrogen‐bonded networks.^[24]^ The higher‐lying six‐ and four‐coordinate complexes, **9‐6 c** and **9‐4 c**, showing a pronounced red‐ and blue‐shift of the Al−H stretch, respectively, are not experimentally observed. At *n=*10, three isoenergetic structures **10‐5 c**‐**a**, **10‐5 c**‐**b**, and **10‐6 c** are considered. Interestingly, in the six‐coordinate structure, the Al−H stretch shifts significantly to the red, explaining the new strong band at around 1870 cm^−1^. As predicted by theory, five‐ and six‐coordinate structures of *n=*10 coexist in the experiment. With one more water molecule, the five‐coordinate infrared signature for *n=*11 has almost disappeared, and the Al−H stretching region is dominated, judging from the broad band, by several six‐coordinate structural isomers with varying wavenumbers, also supported by DFT calculations (Figure S1). At the same time, the absorption at the blue side of the water bending region becomes more intense. For *n=*12, the six‐coordinate structures clearly dominate, and the Al−H stretch shifts slightly to the red and seems to lose intensity. We also probed the O−H stretching region to look for the spectral signature of the hydroxide O−H stretch, but it turns out that this contributes to the broad band of hydrogen‐bonded O−H stretching vibrations, see Figure S3 for the spectrum of *n=*13.

**Figure 3:**
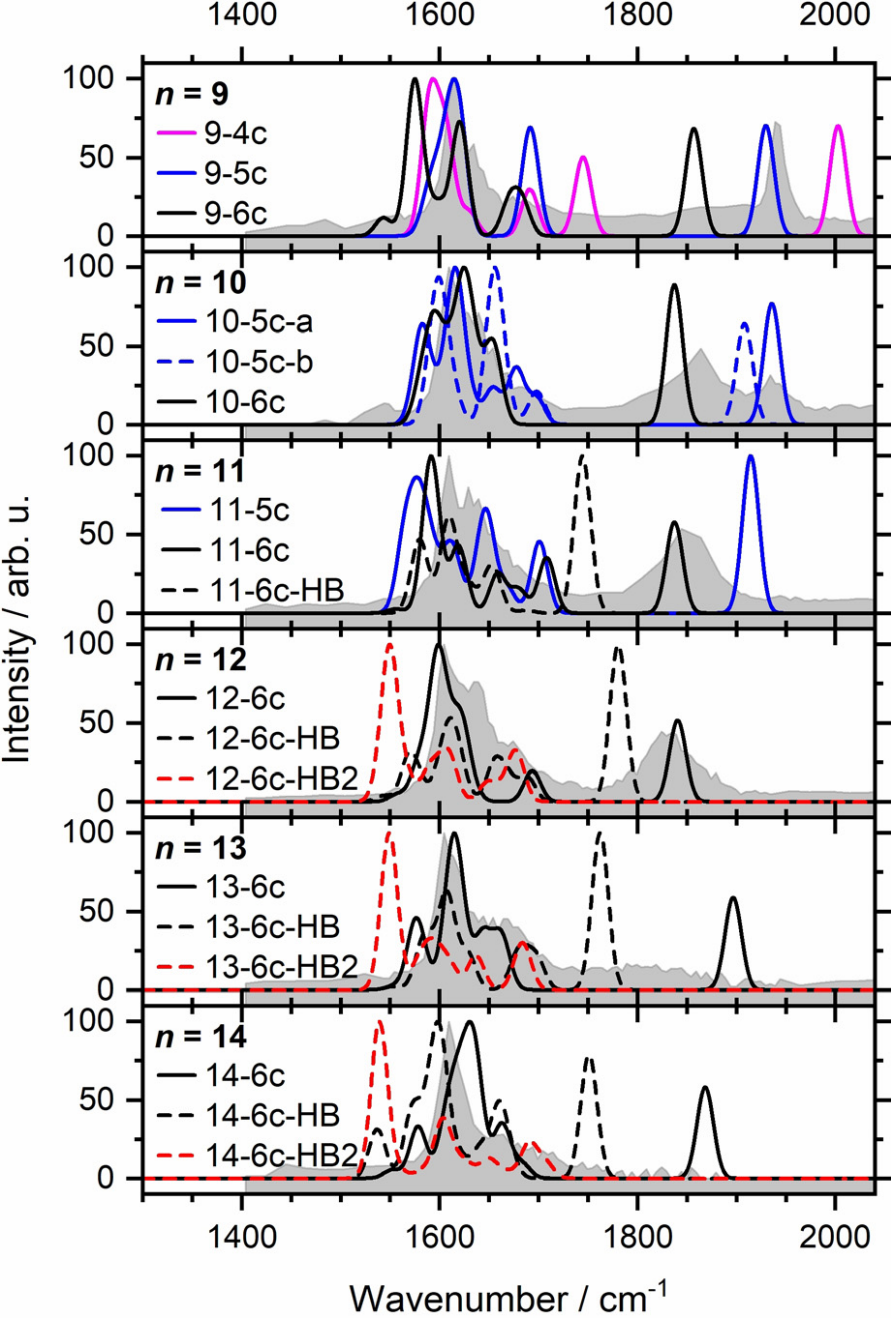
Comparison between experimental IRMPD (gray shaded area) and theoretical IR (lines) spectra calculated at the B3LYP/6‐311++G** level of theory.

It should be noted that at the experimental temperature of 80 K, the onset of the melting transition must be expected for such small clusters.^[25]^ This means that at least one hydrogen bond is broken, providing around 20 kJ mol^−1^ of latent heat. Together with thermally excited vibrations and internal as well as external rotations, one laser IR photon of 18–25 kJ mol^−1^ provides the missing energy for evaporation of a water molecule, calculated as 51–54 kJ mol^−1^, see Table S4. This agrees with our measured IRMPD kinetics, Figure S4, which indicates a one photon process.

Interestingly, structures exhibiting a hydrogen bond towards the hydride become energetically competitive at *n=*11, with a strong red‐shift of the Al−H stretch. The exact position of the Al−H stretch, however, is extremely sensitive to the detailed structure of the hydrogen‐bonded network (Scheme 1). Moreover, double‐acceptor structures like **12‐6 c‐HB2** exhibit an extreme red‐shift. For *n=*13, structures with the hydride acting as a single acceptor can explain the broad absorption between ca. 1720 and 1900 cm^−1^, while the bands of double‐acceptor structures are smeared out below 1580 cm^−1^. With each additional water molecule, the number of energetically accessible cluster isomers increases. At the same time, the position of the Al−H band in the ***n*** **‐6 c‐HB2** structures covers a 200 cm^−1^ spectral range. These two effects together explain why no single strong peak or even band appears in the spectrum that could be assigned to the ***n*** **‐6 c‐HB2** structures.

**Scheme 1:**
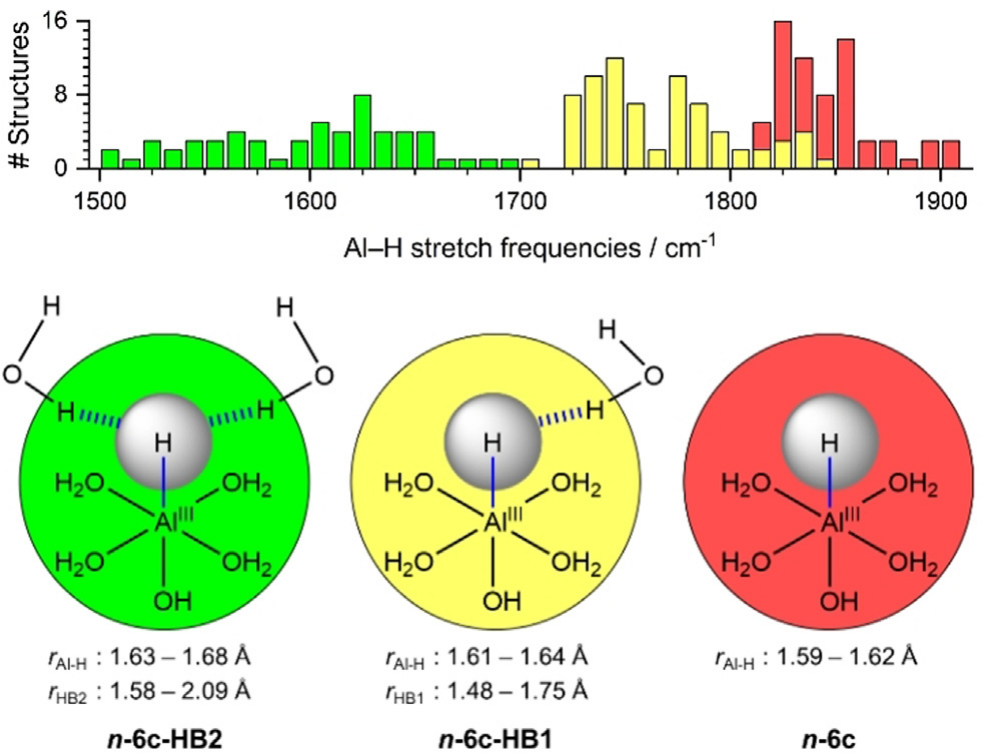
Summary of Al−H stretching frequencies evaluated at the B3LYP/6‐311++G** level. Al−H distance *r*_Al−H_ and water−hydride hydrogen bond distances *r*_HB1_ and *r*_HB2_ were optimized at the M06/6‐311++G** level, including all geometries of six‐coordinate ***n*** **‐6 c**, ***n*** **‐6 c‐HB**, and ***n*** **‐6 c‐HB2** for *n=*11–14. The geometry parameters and Al−H stretch frequencies of all calculated structures are available as Supporting Information, Tables S1–S3. The histogram (top) depicts how many structures were found in each frequency bin.

Usually, a hydrogen bond is formed between a hydrogen atom of the donor and a lone pair of the acceptor, which occupies a sp^3^ hybridized orbital. In the present case, one electron pair in the spherically symmetric s orbital of the hydride acts as the acceptor for two hydrogen bonds, as illustrated in Figure 4. This symmetrical sharing of the acceptor electron pair weakens both hydrogen bonds, evident from their significantly increased lengths in the ***n*** **‐6 c‐HB2** isomers, shown in Scheme 1. The cooperative effects of the hydrogen bonds progressively elongate the Al−H distance ***n*** **‐6 c**<**n‐6 c‐HB** <***n*** **‐6 c‐HB2**, indicating the bond weakening as reflected in the marked redshift.

**Figure 4:**
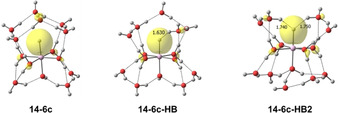
Highest occupied molecular orbitals (HOMO) of **14‐6 c**, **14‐6 c‐HB**, and **14‐6 c‐HB2** with an isovalue of 0.05 a.u. obtained at the M06/6‐311++G** level. The HOMO represents the 1s^2^ electron pair at the hydride which acts as single or double hydrogen bond acceptor.

It is intriguing to note that the appearance of the hydrogen bonded Al−H, which becomes evident in the experiment by the disappearance of the free Al−H stretch, coincides with the onset of H_2_ formation. While structures containing a hydride hydrogen bond can be optimized for the smaller clusters (*n=*9, 10), the respective minima are very shallow and the clusters relax to non‐hydrogen bonded structures when the geometry is slightly distorted. This indicates that at the experimental temperature of 80 K, these structures are not significantly populated, explaining why H_2_ formation is not observed for these sizes. Integration of the hydride into the hydrogen‐bonded network of the cluster is a prerequisite for the hydrogen evolution reaction. Our results strongly support the theoretically predicted H_2_ formation mechanism. Moreover, we show that a metal hydride is a very good hydrogen bond acceptor. Oxygen in water with its two lone pairs can act as double acceptor, while nitrogen, e.g., in ammonia, with only one lone pair is always a single acceptor. Hydride, with two electrons in a spherically symmetric s‐type molecular orbital, can even act as a double acceptor in a hydrogen‐bonded network.

## Conflict of interest

The authors declare no conflict of interest.

## Supporting information

As a service to our authors and readers, this journal provides supporting information supplied by the authors. Such materials are peer reviewed and may be re‐organized for online delivery, but are not copy‐edited or typeset. Technical support issues arising from supporting information (other than missing files) should be addressed to the authors.

Supplementary
